# Association between preoperative fasting time and clinical outcomes in surgical patients in a private general hospital

**DOI:** 10.1590/acb394524

**Published:** 2024-08-16

**Authors:** Rafaela Batista Coutinho, Wilza Arantes Ferreira Peres, Tatiana Pereira de Paula

**Affiliations:** 1 Universidade Federal do Rio de Janeiro – Instituto de Nutrição Josué de Castro – Departamento de Nutrição e Dietética – Rio de Janeiro (RJ) – Brazil

**Keywords:** Preoperative Care, Fasting, Clinical Protocols, Postoperative Complications

## Abstract

**Purpose:**

Surgical patients are routinely subjected to long periods of fasting, a practice that can exacerbate the metabolic response to trauma and impair postoperative recovery. The aim of this study was to evaluate the association between preoperative fasting time and clinical outcomes in surgical patients.

**Methods:**

An observational, prospective study with a non-probabilistic sample that included patients of both sexes, aged over 18, undergoing elective surgeries. Data were extracted from electronic medical records, and a questionnaire was applied in 48 hours after surgery. Variables related to postoperative discomfort were assessed using an 11-point numeric rating scale.

**Results:**

The sample consisted of 372 patients, and the duration of the surgical event ranged from 30–680 minutes. The incidence of nausea (26.34%) was twice that of vomiting (13.17%) and showed an association with the surgical procedure’s size (*p* = 0.018). A statistically significant difference was observed only between pain intensity and preoperative fasting times for liquids (*p* = 0.007) and postoperative fasting time (*p* = 0.08). The occurrence of postoperative complications showed no association with preoperative fasting time (*p* = 0.850).

**Conclusions:**

Although no association was observed between preoperative fasting time and surgical complications, it is noteworthy that both recommended and actual fasting time exceeded the proposed on clinical guidelines.

## Introduction

The practice of preoperative fasting gained prominence in the mid-20th century following a study published by Mendelson (1946), which recommended fasting as a measure to prevent post-anesthetic respiratory complications resulting from the aspiration of gastric contents into the lungs^1^^,^ 2. The change from this fasting pattern to current guidelines was initially proposed in 1999 by the American Society of Anesthesiologists (ASA)^3^.

In Brazil, inspired by fast-track protocols from the 1980s and Enhanced Recovery After Surgery (ERAS), the Acceleration of Total Postoperative Recovery (ACERTO) project, originally conceived as a research project, was implemented in 2005 in the surgical ward of the Júlio Muller University Hospital (Cuiabá, MT, Brazil), following a six-month audit. Based on evidence-based practice, since its inception, ACERTO has expanded the vision of perioperative care by implementing new multimodal conducts to different surgical specialties, clinical profiles, and surgical sizes^4^.

Prolonged preoperative fasting is a harmful and unnecessary process for most patients, which can exacerbate the cascade of the metabolic response to trauma, worsen insulin resistance and protein catabolism, and alter gastrointestinal function^5^. Moreover, other physiological and psychosocial effects associated with patient discomfort, such as thirst, hunger, dehydration, electrolyte imbalance, increased postoperative nausea and vomiting, irritability, headache, and anxiety^6^.

Several anesthesia societies and medical organizations have advocated for more liberal preoperative fasting periods, ranging from 2 to 8 hours, depending on the type of food consumed: 2 hours for clear liquids enriched with carbohydrates; 4 hours for breast milk; 6 hours for light meals, enteral diets, infant formulas, and non-human milk; and 8 hours for solid foods, including large meals containing meats, fried foods, and fatty preparations^7^^–^ 10.

The abbreviation of preoperative fasting with the administration of carbohydrate-enriched solutions up to 2 hours before anesthesia induction, in addition to not causing harm, has been associated with positive clinical outcomes, such as reducing the organic response to surgical stress, lower insulin resistance, improved well-being, reduced irritability, especially in children; reduced nausea/vomiting, increased pH, better gastric emptying, cost reduction, and shorter hospital stays^11^^–^ 14.

This study aimed to verify the association between preoperative fasting time and clinical outcomes in patients undergoing elective surgeries.

## Methods

### Study design

This is an observational, analytical and prospective study conducted at a tertiary hospital located in Campos dos Goytacazes, RJ, Brazil, which annually performs over 5,000 elective and emergency surgeries across different specialties. During the period of this study, the unit did not have a preoperative fasting abbreviation protocol. Eligibility criteria included patients of both sexes, aged over 18 years, undergoing elective surgeries between February and December 2022. Patients under 18 years old, those undergoing emergency surgeries, individuals with esophageal obstruction, intestinal obstruction, symptomatic gastroesophageal reflux, chronically decompensated diabetes *mellitus*, megaesophagus, gastroparesis, pyloric stenosis, or other conditions causing delayed gastric emptying were not included.

The sample was non-probabilistic and convenience-based, consisting of individuals who voluntarily agreed to participate in the study by signing an informed consent form. The study was planned in accordance with the ethical aspects outlined in Resolution no. 466/2012 of the National Health Council, and it was reviewed and approved by the Research Ethics Committee of Clementino Fraga Filho University Hospital/Universidade Federal do Rio de Janeiro (Certificate of Presentation of Ethical Review no. 50359321.1.0000.5257).

### Variables

Information regarding sex, age, comorbidities, total length of hospital stay, type and size of surgery, ASA risk score, type of anesthesia technique, and time of anesthetic induction were extracted from electronic medical records.

The ASA risk score is a classification of the patient’s physical status, categorizing them based on their overall clinical condition and the presence or absence of systemic disease^15^. In addition to surgical size reported in the medical records by the surgical team, surgeries were categorized into two sizes, according to duration: minor, for surgeries with the duration of 4 hours or less, and major, for surgeries lasting more than 4 hours^16^.

Patients were followed in a hospital setting for a period of 90 days, and the following variables were also considered: mortality, frequency of hospital readmission, frequency of surgical re-intervention, and occurrence of perioperative complications (aspiration, wound dehiscence, evisceration, abscess or wall collection, active bleeding from surgical wound, suture or anastomosis dehiscence and fistula, cavitary abscess or collection, inadvertent injury to structure(s), cavitary bleeding, surgical site infection, and prolonged ileus).

### Data collection

Data collection was conducted in 48 hours after the surgical event by applying a questionnaire to the patient. The questionnaire aimed to collect information about the time of pre-procedure hospitalization, preoperative fasting guidance and recommended time, actual preoperative fasting time, postoperative fasting time until resumption of oral or enteral feeding, and postoperative discomfort.

Preoperative fasting time was calculated based on the time of the last intake of solids and liquids and the time of anesthetic induction. Postoperative fasting time was considered the time difference between the end of surgery and the introduction of oral or enteral feeding. Total fasting time was defined as the time elapsed between the time of the last intake of solids and the introduction of oral or enteral feeding after surgery.

Variables related to postoperative discomfort, such as pain, hunger, and thirst, were assessed using an 11-point numeric rating scale (NRS) (0 = absence of symptom; 10 = extreme symptom), and nausea/vomiting were assessed based on occurrence and number of episodes. The NRS is a unidimensional instrument that classifies symptom intensity based on self-report by individuals. It is a simple, quick, easily applicable, and cost-effective tool validated for various types of patient and has demonstrated good performance for pain intensity assessment. Scores are interpreted as: 0 = absence of symptoms, 1–3 = mild symptoms, 4–6 = moderate symptoms, and 7–10 = intense symptoms^17^^,^ 18.

### Statistical analysis

All statistical analyses were performed using the Statistical Package for the Social Sciences software, version 22 for Windows (SPSS, Inc., Chicago, IL, United States of America). Data distribution and adherence to the normal curve were verified using the Kolmogorov-Smirnov Test.Continuous variables were described using means and their respective standard deviations, while non-parametric variables were described using medians and interquartile range (IQR), as appropriate.. Categorical variables were expressed as absolute numbers and relative frequencies. Data distribution and adherence to the normal curve were verified using the Kolmogorov-Smirnov’s test, and non-parametric tests were adopted based on the results. Associations between categorical variables were tested using the χ^2^ test. For the comparison of numerical variables between two groups with independent samples, the Mann-Whitney’s test was used, while for comparisons among three or more groups the Kruskal-Wallis’ test was applied. The Dunn multiple comparisons test was used to identify differences between subgroups. The level of statistical significance was set at 5% (p < 0.05).

## Results

The sample consisted of 372 patients, with 59.14% (n = 220) being female and mean age of 52.40 ± 17.23 (18–95). One hundred and six patients (28.49%) were transferred to the intensive care unit (ICU) immediately postoperatively, with a median length of stay in this unit of two days (IQR = 1;2). General anesthesia was the anesthetic technique used in 69.95% (n = 256) of the surgeries. Forty-four patients (49.44%) had an ASA risk score of I, and 75% (n = 147) underwent major surgeries. The duration of the surgical event ranged from 30 to 680 minutes, with 52.42% (n = 195) of the surgeries performed in 120 minutes (Table 1). Oncological surgeries were the most common (30.91%), followed by gastrointestinal (16.57%) and plastic surgeries (15.32%) (Table 2).

**Table 1:** Description of sociodemographic data, perioperative routine, and characteristics of elective surgeries performed at a tertiary hospital.

Variables	Frequency
**Age (years old)**	
Mean ± standard deviation	52.40 ±17.23
**Sex**	
Female	220 (59.14%)
Male	152 (40.86%)
**LHS (days)**	
Median (IQR)	2 (1-4)
**LHS post-operative (days)**	
Median (IQR)	2 (1-2)
**Days of post-operative intensive care unit**	
No	266 (70.51٪)
Yes	106 (28.49٪)
Median (IQR)	2 (1-2)
**ASA (n = 89)** ¥	
I	44 (49.44٪)
II	40 (44.94٪)
III	5 (5.62٪)
Surgical size* (n=196) ¥	
Minor	3 (1.53٪)
Medium	46 (23.47٪)
Major	147 (75٪)
Surgical size versus time**	
Minor (≤ 4h)	302 (81.18٪)
Major (> 4h)	70 (18.82٪)
**Duration of surgery (minutes)**	
Median (IQR)	135 (85–240)

¥ Data with incomplete records in the analyzed medical records;

LHS: length of hospital stay; IQR: interquartile range;

* surgical size described in the medical records;

** surgical size defined according to duration of the procedure;

ASA: American Society of Anesthesiologists. Source: Elaborated by the authors.

**Table 2:** Distribution types of elective surgeries performed at a tertiary hospital.

Type of surgery	N (%)
Oncology	115 (30.91%)
Plastic	57 (15.32%)
Abdominal	44 (11.83%)
Orthopedic	37 (9.95%)
Gastrointestinal Tract	33 (8.87%)
Neurological	21 (5.65%)
Urological	21 (5.65%)
Vascular	12 (3.23%)
Cardiac	11 (2.96%)
Gynecological	7 (1.88%)
Otorhinolaryngology	5 (1.34%)
Thoracic	5 (1.34%)
Others*	4 (1.07%)

* Maxillary and ophthalmological.

Source: Elaborated by the authors.

The preoperative fasting time for solids ranged from 5 hours and 50 minutes to 65 hours and 10 minutes, with 64.25% (n = 239) of patients abstaining from solid food for 12 hours or more. Almost 45% (n = 166) did not consume liquids for an equal period, 39.52% (n=146) had their diet resumed in 4 hours after the surgical event, and 40.34% (n = 150) underwent total fasting for 24 hours or more. The medians for the actual preoperative fasting time for solids and liquids, postoperative fasting time, and total fasting time were 13.54 (IQR = 11–17) hours, 11.37 (IQR = 9.33–14.67) hours, 4.67 (IQR = 3.17–12.85) hours, and 22 (IQR = 17.62–29) hours, respectively. Abdominal surgeries had a higher median fasting time for solids, 15.25 (IQR = 13–17.94) hours. Oncological surgeries had a shorter fasting time for liquids, 9.42 (IQR = 8.38–14.79) hours. Only in cardiac and vascular surgeries 100% of the patients received a diet in 24 hours postoperatively.

Regarding the consistency/type of diet offered in the first postoperative meal, 36.83% (n = 137) received a soft diet, 16.13% (n = 60) received a clear liquids diet, and 2.96% (n = 12) received a diet by enteral route. During hospitalization, two patients received parenteral nutrition due to postoperative complications.

**Figure 1:**
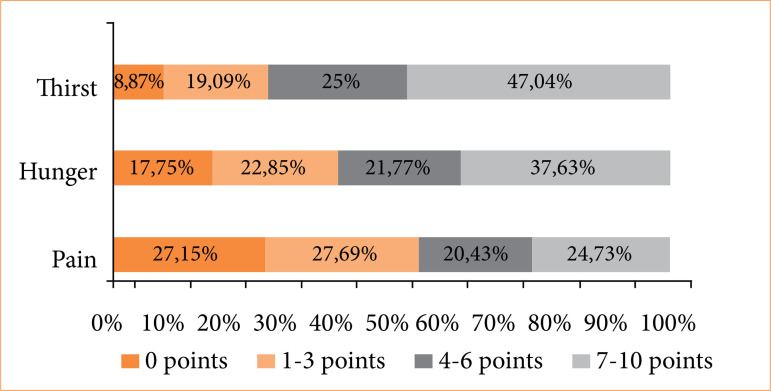
Description of the intensity of variables related to discomfort (thirst, hunger, and pain) assessed using an 11-point numeric rating scale in patients undergoing elective surgeries (n = 372).

Source: Elaborated by the authors.

Thirst had the highest score among the three variables analyzed using the NRS, with a median of 6 (IQR = 3–9) points, as shown in Fig. 1. Nausea was reported by 26.34% (n = 98) of participants, and vomiting by 13.17% (n = 49).

Patients who experienced longer preoperative fasting times for solids and liquids reported intense pain, while those with longer times for diet reintroduction postoperatively mentioned moderate pain (Table 3). There was a statistically significant difference only between pain intensity and preoperative fasting times for liquids (*p* = 0.007) and diet reintroduction time postoperatively (*p* = 0.08). Preoperative fasting time for liquids and time for diet reintroduction were longer among patients reporting intense pain (*p* = 0.005) and moderate pain (*p* = 0.004), respectively.

**Table 3:** Comparison between preoperative fasting time for solids and liquids, diet reintroduction time, and total perioperative fasting time in hours, and the intensity of pain in patients undergoing elective surgeries!

Pain	Preoperative fasting (solids)	Preoperative fasting (liquids)	Postoperative fasting	Perioperative fasting
Absent	13.7 (11–16)	11.4 (8.8–14)a,b	3.83 (2.8–8.58)^a^	21.33 (17.5–26)
Mild	12.7 (10.92–17)	10.6 (8.72–13.05) a	4.67 (3.1–11.6)a,b	21.5 (17.7–26.5)
Moderate	13.1 (11–18)	11.5 (9.93–14.67)a,b	6.17 (4.3–16.5)^b^	22.5 (17.2–35.2)
Intense	14.8 (11.2–17.7)	13 (10–16.5)^b^	5 (3.3–13.35)a,b	21.5 (16.9–27.5)
*p-value*	0.543	0.007*	0.008*	0.714

! Results: median (interquartile range) in hours.

Data was compared by the Kruskal-Wallis’ test;

* *p* < 0.05;

a,b medians followed by the same letter do not differ, distinct letters indicate statistical difference at the 5% significance level.

Source: Elaborated by the authors.

The results indicated that the surgical size (minor or major according to time) influenced the occurrence of nausea (*p* = 0.023); a major surgery was associated with a higher incidence of this symptom. However, no such correlation was observed between surgical size and the occurrence of vomiting (Table 4).

**Table 4:** Association between surgical size and the occurrence of postoperative nausea and vomiting in patients undergoing elective surgeries*.

Symptoms	Surgical size		
	Minor (≤ 4 h)	Major (> 4 h)	*p*-value
**Nausea**			
Yes (n = 98)	72 (73.5%)	26 (26.5%)	0.023
No (n = 274)	230 (83.9%)	44 (16.1%)	
**Vomiting**			1 mL
Yes (n = 49)	38 (77.6%)	11 (22.4%)	0.485
No (n = 323)	264 (81.7%)	59 (18.3%)	

* Data compared using the χ^2^ test.

Source: Elaborated by the authors.

There was no association between the occurrence of nausea and perioperative fasting times, neither between the occurrence of vomiting and preoperative fasting times for solids and liquids. However, it was observed that the time for diet reintroduction postoperatively (*p* = 0.021) and the total fasting time (*p* = 0.002) were significantly longer in the group of patients who reported postoperative vomiting episodes.

Among the adverse events in 90 days and perioperative complications, there were cases of surgical wound infection (n = 6), fistulas (n = 4), wall abscess (n = 3), prolonged ileus (n = 1), suture dehiscence (n = 1), cavitary collection (n = 1), as well as the need for hospital readmission (n = 13) and surgical re-intervention (n = 9). There was no record of pulmonary aspiration, but one patient experienced mortality. The occurrence of postoperative complications showed no association with preoperative fasting time (*p* = 0.850).

## Discussion

The results found in this study contradict the recommendations for fasting abbreviation. The fasting time was 13.54 (IQR = 11–17) hours for solids, 11.37 (IQR = 9.33–14.67) hours for liquids, 4.67 (IQR = 3.17–12.85) hours until diet reintroduction and 22 (IQR = 17.62–29) hours in the perioperative period. In Cestonaro et al.’s study^19^, the preoperative fasting time for solids and liquids and postoperative fasting time were similar, respectively, 16.50 (5.50–56.92) hours; 15.75 (2.50–56.92) hours, and 15.67 (1.67–90.42) hours. Over 90% of participants fasted for solids and liquids for over 9 hours, and 60% received their first meal 10–20 hours after surgery.

In a multicenter cohort study with 924 patients, Beck et al.^20^ observed an average preoperative fasting duration of 17.02 ± 6.54 hours for solid foods and 9.21 ± 5.48 hours for liquids, nearly five times longer than recommended by guidelines. Solid food reintroduction occurred after 9.42 ± 12 hours after surgery, and the mean perioperative fasting time for solids was 28.23 ± 14.02 hours^20^. Zhu et al.^21^ found that patients undergoing elective surgeries at a tertiary hospital also had a high average fasting time for solids, 13.41 ± 2.64 (7–20) hours, and for liquids, 10.27 ± 3.67 (1.5–18) hours.

Diet reintroduction occurred in 24 hours of surgery in 91.67% of cases, following recommendations of starting a diet in the first 24 hours postoperatively, provided the patient is hemodynamically stable^9^. Early postoperative nutrition is not only safe, but also reduces the risk of anastomosis dehiscence, lowers postoperative complications, shortens hospital stays, reduces hospital costs, and decreases morbidity and mortality^9^^,^ 22 ^,^ 23.

Dehydration, hypoglycemia, a higher incidence of postoperative nausea and vomiting, and increased patient discomfort (*e.g.*, hunger, thirst, fatigue, and anxiety) are some of the negative physiological effects associated with prolonged fasting^24^^,^ 25. Regarding the intensity of postoperative discomfort symptoms, there was mild pain (3; IQR = 0–6 points), moderate thirst (6; IQR = 3-9 points), and moderate hunger (6; IQR = 0-6 points). No thirst, hunger and pain were reported by 8.9, 17.7, and 27.2% of patients, respectively.

Gul et al.^25^ analyzed the relation between traditional fasting policies and preoperative discomfort. About 80% of patients reported no thirst or hunger on the night before surgery, but symptoms intensified with prolonged fasting. On the day of surgery, 43.3% of participants felt some degree of hunger or thirst, 46.4% reported mild to intense weakness, and 39.6% experienced mild discomfort. Furthermore, preoperative fasting time for solids was associated with thirst, hunger, dry mouth, and weakness, while total fluid fasting time was associated with hunger and dry mouth.

A meta-analysis involving 5,606 patients from 57 randomized controlled trials that aimed to evaluate the effect and safety of carbohydrate intake in the preoperative period of adult surgical patients observed that this practice reduces discomfort, including dry mouth, thirst, hunger, and pain; reduces hospitalization time; and decreases insulin resistance when compared to fasting. Given the benefits, safety, and low cost of offering carbohydrates preoperatively, the authors suggested that this strategy should be adopted by surgeons and anesthesiologists to improve surgical recovery^26^.

Pain is reported as the primary complication in the postoperative period. In this study, the preoperative fasting time for liquids and the postoperative fasting time were longer among patients who reported intense pain (*p* = 0.005) and moderate pain (*p* = 0.004), respectively. Torabikhah et al.^27^, when investigating the impact of reducing the preoperative fasting time on postoperative pain in orthopedic patients, found that the pain intensity in the group with shortened fasting was lower than that in the control group, but without statistical significance. The authors considered that the subjectivity of the concept of pain and the lack of a more precise tool for measuring this symptom may have influenced the results. It is likely that a shorter fasting time allows patients to be more relaxed and have a lower perception of pain, minimizing discomfort^28^.

Nausea and vomiting are undesirable, but preventable effects that lead to dissatisfaction and complications such as dehydration, electrolyte imbalance, wound dehiscence, and delays in hospital discharge^29^. The overall incidence of postoperative nausea and vomiting (PONV) is 50 and 30%, respectively^30^, higher than the findings in this study, in which 26.34% of patients reported nausea and 13.17% reported vomiting. Marquini et al.^31^ randomized 80 women undergoing gynecological surgeries into two groups (control group: 200 mL of inert solution; intervention group: 200 mL of a clear supplement 4 hours before the procedure) to assess the effects of preoperative fasting abbreviation on the incidence of PONV. The incidence of nausea and vomiting was also lower than described in the literature, with 18.9% in the control group and 10.8% in the intervention group, but there was no statistically significant difference between the groups.

Among the independent risk factors for developing PONV are female gender, history of postoperative nausea and vomiting or motion sickness, non-smoking status, and postoperative opioid use^29^^–^ 31. The association between the type of surgery and PONV is still not well defined, but it is believed that longer surgeries favor its occurrence due to prolonged exposure to general anesthesia and high-dose opioid administration. PONV prophylaxis is indicated for all surgical patients and involves reducing preoperative fasting time, multimodal analgesia strategies, conscious fluid use, and antiemetic use for patients at medium or high risk^32^^,^ 33.

Xu et al.^34^ revealed through a meta-analysis that reducing preoperative fasting time for patients undergoing laparoscopic cholecystectomy increases postoperative comfort by reducing the incidence of nausea and vomiting, improving insulin resistance, and minimizing the stress response. A study with patients undergoing elective colorectal surgeries using measures proposed by the ACERTO protocol analyzed risk factors associated with various clinical outcomes and demonstrated, through univariate analysis, that prolonged preoperative fasting time (greater than 4 hours) increased the risk of surgical site infection by more than five times, anastomotic fistula by 9.27 times, pneumonia-atelectasis by 10 times, and death by 20 times^35^.

In the present study, 6.18% of patients had postoperative complications. Although high, the preoperative fasting duration (13.54; IQR = 11; 17 hours) evidenced no association with postoperative complications (*p* = 0.850). The sample size may have hindered the accurate statistical assessment of the association between fasting time and the occurrence of postoperative complications, a situation attributed to type II error. Lucchesi and Gadelha^36^ identified surgical wound infection (27.2%) and surgical re-intervention (18.2%) as the most frequent complications and emphasized that there was no significant difference between nutritional status or perioperative fasting time and the observed complications^36^. Virgens et al.^37^ found, in a sample of gastric and colorectal cancer surgical patients, that prolonged perioperative fasting time (59.0 ± 2.4 hours) was an independent predictor of length of stay, but it was not associated with the incidence of postoperative complications and death.

This study has some limitations. Many medical records had incomplete information, making it impossible to properly collect all proposed study variables. Postoperative patient blood glucose levels were not collected; this data could complement the assessment of metabolic complications associated with prolonged fasting. On the other hand, the results found in this research indicate the need for remodeling perioperative protocols, such as the implementation of a preoperative fasting abbreviation protocol, and may direct educational and training actions for teams.

## Conclusion

Traditional preoperative fasting practices are still commonly followed, despite extensive literature supporting the safety and benefits of fasting abbreviation. In the studied sample, preoperative fasting times exceeded guideline recommendations, particularly fasting time for liquids, which was almost six times longer than recommended. The preoperative fasting time for liquids and the postoperative fasting time were related to the intensity of postoperative pain.

The occurrence of nausea was influenced by the surgical size, but it did not impact the time of perioperative fasting; on the other hand, the occurrence of vomiting showed a significant association with a longer time to reintroduce the diet postoperatively and with the total fasting time. There was no difference between prolonged fasting and postoperative complications, or with thirst, the main discomfort complaint reported by patients.

The results of this study highlight the need for the implementation of a preoperative fasting abbreviation protocol, as well as the development of actions to ensure its execution to improve the safety and care of surgical patients.

## Data Availability

All data sets were generated or analyzed in the current study.
